# Secondary syphilis occurring under anti-CD20 therapy: risks, progression and approach^⋆^

**DOI:** 10.1016/j.abd.2023.07.006

**Published:** 2024-04-12

**Authors:** Luiz Augusto Fabricio de Melo Garbers, Tissiana Marques de Haes, Cacilda da Silva Souza

**Affiliations:** a Department of Internal Medicine, Faculdade de Medicina de Ribeirão Preto, Universidade de São Paulo, Ribeirão Preto, SP, Brazil; b Department of Neurosciences and Behavioral Sciences, Faculdade de Medicina de Ribeirão Preto, Universidade de São Paulo, Ribeirão Preto, SP, Brazil

Dear Editor,

The increasing use of immunotherapies has raised questions regarding changes in the immune response to syphilis in patients receiving these therapies.

A 22-year-old man using ocrelizumab (anti-CD20) for multiple sclerosis, presented with a non-pruritic skin rash and two febrile episodes 20 days before consultation. When questioned, he mentioned scaly lesions on his hands; erythema and desquamation, without ulceration on the glans; the lesions resolved spontaneously, approximately four weeks after unprotected sexual intercourse. On examination, erythematous-coppery maculopapular lesions were observed on the trunk, some with a central hematic crust on the upper part (Fig. 1A,B); finely scaly lesions surrounding the forehead, temporal, periauricular, and posterior cervical regions, in the shape of a crown (corona veneris) (Fig. 2), and with exuberant peripheral desquamation (Biett collarette) on the palms of the hands, soles of the feet and wrists (Fig. 3A,B). These cutaneous and systemic manifestations indicated secondary syphilis. The laboratory investigation showed a low serum titer in the VDRL (Venereal Disease Research Laboratory) test (1/2), and negativity in the cerebrospinal fluid, with hyperproteinorrhaquia, 56 mg/dL (15‒45 mg/dL); a positive confirmatory chemiluminescence test for treponemal antibodies, and a negative test for human immunodeficiency virus (HIV). The IgM measurement was reduced, 24 mg/dL (50‒300 mg/dL), and IgA and IgG were normal. Intramuscular benzathine penicillin was recommended, 2.4 million units weekly, for three consecutive weeks, followed by intravenous ceftriaxone, 2 g daily for 14 days.

**Figure 1:**
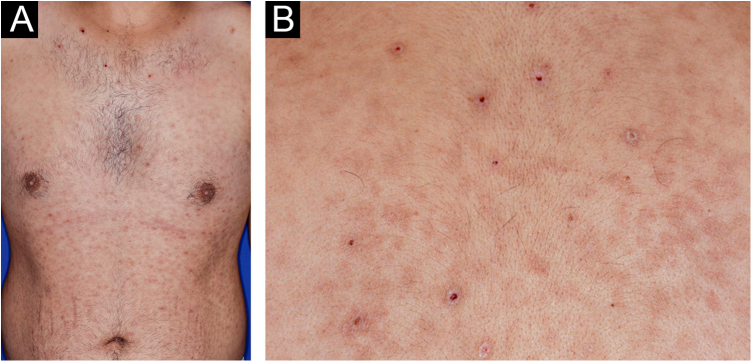
(A) Erythematous/copper-colored macules and papules on the trunk; (B) Lesions with overlapping central hematic crust on the upper part.

**Figure 2:**
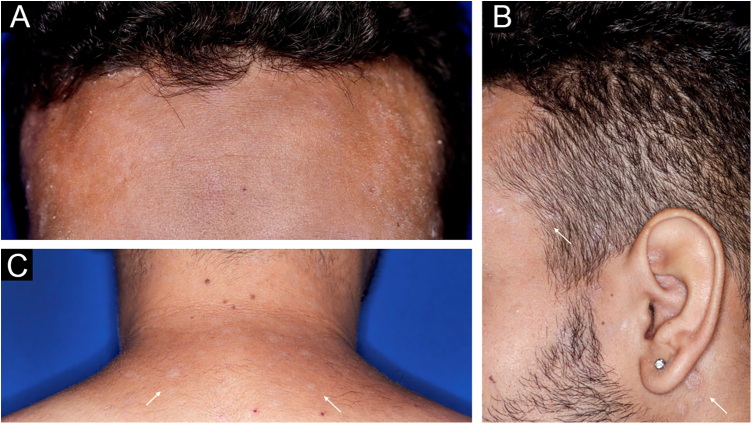
(A) *Corona veneris*: thin peripheral desquamation (Biett’s collarette) over lesions surrounding the forehead, (B) temporal, pre-, intra- and retroauricular regions and (C) base of the neck (white arrows).

**Figure 3:**
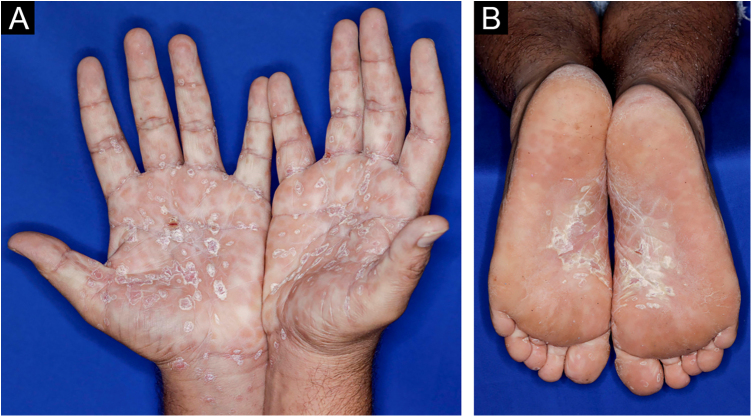
(A) Exuberant Biett’s collarette over erythematous maculopapular lesions on the palms, wrists, and (B) soles of feet.

*Treponema pallidum* penetrates through microabrasions in mucous membranes or skin, genital or extragenital, forming a painless ulcer with a hardened base (primary chancre). Six to ten weeks after the primary infection, secondary syphilis manifests as a polymorphic skin and/or mucosal eruption, lymphadenopathy, and other nonspecific systemic symptoms.

Serological non-treponemal screening tests (VDRL, RPR ‒ Rapid Test Reagin) and confirmatory treponemal tests (FT-ABS, TPHA ‒ Treponema Pallidum Hemagglutination Assay, TPPA ‒ Treponema Pallidum Particle-Agglutination assay) are widely used for the diagnosis of syphilis, and the molecular PCR assay and immunohistochemistry should be considered in the investigation of atypical cases, or conditions in which the humoral response and serological tests may be impaired.^1^, ^2^

Ocrelizumab and rituximab are monoclonal antibodies against the CD20 marker on B lymphocytes. Their depletion involves a risk of decreasing immunoglobulin levels and, consequently, the humoral response to neoantigens. After anti-CD20 therapy, B cell recovery is slow and non-uniform, and memory B cells may remain depleted. Compared to ocrelizumab, rituximab was an independent predictor of hypogammaglobulinemia, which may reduce the humoral response.^1^

A previous report of a patient treated with rituximab for multiple sclerosis and skin eruption typical of secondary syphilis showed negative VDRL and TPPA serological tests on repeated measurements and delayed seroconversion for more than three weeks after the onset of symptoms and skin manifestations, but PCR and immunohistochemistry were positive for *T. pallidum*.^2^

Reduced humoral response resulting in a delayed seroconversion, low titer or even negative serological tests for syphilis should be expected in patients receiving anti-CD20 class therapy. These conditions can make diagnosis difficult, and clinical manifestations become crucial indicators for suspicion and persistence in the investigation of syphilis, with confirmatory immunohistochemical or molecular tests, and possible involvement of other systems.

In addition to the six cases in the Iglesias-Plaza review,^3^ seven more cases of syphilis occurring in patients receiving immunobiological therapy were identified. Most of them were male (9), aged between 16 and 66 years, with psoriasis and/or psoriatic arthritis (4), rheumatoid arthritis (2), juvenile idiopathic arthritis (1), ankylosing spondylitis (1), Chron’s disease/ulcerative rectocolitis (2) and multiple sclerosis (1); using infliximab (3), adalimumab (3), etanercept (2), ustekinumab (1), golimumab (1), tocilizumab (1), secukinumab (1) and rituximab (1), according to the respective indications. Among seven investigated cases (7/13), five cases (71.43%) had confirmation of neurosyphilis, and three were symptomatic. All patients on anti-TNF therapy had positive serological tests; however, immunosuppression is a risk factor for the accelerated course of the disease, and potentially for neurosyphilis (Supplementary Table 1).^3^, ^4^, ^5^, ^6^, ^7^, ^8^ The confirmation of neurosyphilis, ocular syphilis or otosyphilis requires the intravenous use of crystalline penicillin G 18 to 24 million units per day, or alternatively ceftriaxone 1 g to 2 g daily (intravenously or intramuscularly) for ten to 14 days.^9^

Risk reduction strategies in immunocompromised patients include education and preventive counseling for sexually transmitted infections, in addition to screening and routine serology for immunobiological use.

Syphilis mimicry can bring difficulties for diagnostic suspicion, and there can be rapid progression and challenges in confirming the etiology in those with immunosuppression, requiring meticulous clinical examination, careful laboratory investigation and interpretation, well as the use of additional diagnostic methods.

## Financial support

None declared.

## Authors’ contributions

Luiz Augusto Fabricio de Melo Garbers: data acquisition, collection, or analysis and interpretation of data; drafting and editing of the manuscript; intellectual participation in the propaedeutic and/or therapeutic conduct of the studied case; approval of the final version of the manuscript.

Tissiana Marques de Haes: Data acquisition, collection, or analysis and interpretation of data; intellectual participation in the propaedeutic and/or therapeutic conduct of the studied case; approval of the final version of the manuscript.

Cacilda da Silva Souza: Design and planning of the study; data acquisition, collection, or analysis and interpretation of data; drafting and editing of the manuscript or critical review of the intellectual content; intellectual participation in the propaedeutic and/or therapeutic conduct of the studied case; critical review of the literature; approval of the final version of the manuscript.

## Conflicts of interest

None declared.
