# Supplementary material for: Secondary syphilis occurring under anti-CD20 therapy: risks, progression and approach
Source: An Bras Dermatol. 2024 Apr 12;99(4):632–4. doi: 10.1016/j.abd.2023.07.006 (PMC11221156; doi:10.1016/j.abd.2023.07.006)
Supplement: Supplementary file 1 [file mmc1.docx]

ABD-D-23-00293_Supplementary Material

**Supplementary  Table 1** Demographic, clinical and laboratory data of patients with syphilis using immunobiologicals in publications in the English-language literature, between 2010 and 2021.

| **n** | **Reference (year)** | **Age (years), Sex** | **Disease** | **Medication (class)** | **Diagnostic Hypotheses** | **Neurological symptoms** | **Serological tests** | **Tests in cerebrospinal fluid** | **Treatment** |
| --- | --- | --- | --- | --- | --- | --- | --- | --- | --- |
| 1 | Bories-Haffner et al. (2010) | 35, F | Ankylosing spondylitis | Etanercept/Infliximab (anti-TNF) | Pityriasis rosea, Mycosis fungoides | No | VDRL (1/128) | VDRL (1/32) TPHA (1/320) | C-PenG 24 M IU, IV, 15 days |
|  |  |  |  |  |  |  | TPHA (1/20,480) |  |  |
| 2 | Asahina et al. (2012) | 56, F | Rheumatoid arthritis | Etanercept/ Adalimumab (anti-TNF) | Paradoxical skin reaction | No | TPHA (1/10,240) | No information | Oral amoxicillin (dose/duration not specified) |
| 3 | Bettenworth et al. (2012) | 37, M | Ulcerative colitis | Infliximab (anti-TNF) |  | Yes, exacerbation of ankle reflexes | VDRL (+) | TPPA (1/32) | C-PenG 10 M IU, IV, 14 days |
|  |  |  |  |  |  |  | TPPA (+) |  |  |
|  |  |  |  |  |  |  | FTA-ABS (+) |  |  |
| 4 | Assikar et al. (2013) | 62, M | Ankylosing spondylitis | Etanercept (anti-TNF) |  | Yes, hypomania episodes | VDRL (1/180) | Negative VDRL, lymphocytosis | C-PenG 14 days (dose not specified) |
|  |  |  |  |  |  |  | TPHA (1/1,280) |  |  |
| 5 | Kase et al. (2014) | 40, M | PsO | Infliximab (anti-TNF) |  | Yes, emotional lability and excitability | RPR (+) | RPR (+) | C-PenG 24 M IU, IV, 9 days and Ceftriaxone, 28 days |
|  |  |  |  |  |  |  | TPPA (+) | TPPA (+) |  |
| 6 | Pedrosa et al. (2016) |  |  |  |  |  |  |  |  |
|  | Case 1 | 54, M | PsO/PSA | Etanercept (anti-TNF) |  | No information | VDRL (1/64) | No information | B-PenG 2,4 M IU, IM, single dose |
|  |  |  |  |  |  |  | TPPA (+) |  |  |
|  |  |  |  |  |  |  | PCR (+) |  |  |
|  | Case 2 | 66, M | PsO | Adalimumab (anti-TNF) |  | No information | VDRL (1/512) | No information | B-PenG 2.4 M IU, IM, weekly, three consecutive weeks |
| 7 | Uslu et al. (2017) | 19, M | PsO/PSA | Ustekinumab (anti-IL12/23) | PsO relapse; superficial mycosis | No information | TPHA (1/640) | No information | Ceftriaxone 2g daily, IV, 18 days |
|  |  |  |  |  |  |  | FTA-ABS (+) |  |  |
| 8 | Iglesias-Plaza et al. (2019) | 30, M | Ankylosing spondylitis | Golimumab (anti-TNF) |  | No | VDRL (+) | Negative RPR | B-PenG 2.4 M IU, IM, weekly, three consecutive weeks |
|  |  |  |  |  |  |  | RPR (+) |  |  |
|  |  |  |  |  |  |  | FTA-ABS (+) |  |  |
| 9 | Yıldızhan et al. (2019) | 29, F | Crohn's disease after gastrectomy | Adalimumab (anti-TNF) | PG, Disseminated deep mycosis, PLEVA, generalized HZ, LP, PCTCL | No information | VDRL (1/16) | No information | B-PenG 2.4 M IU, IM, weekly, three consecutive weeks |
| 10 | Dos Santos et al. (2020) | 16, F | Juvenile idiopathic arthritis | Tocilizumab (anti-IL6) |  | No | VDRL (1/32) | Negative VDRL | B-PenG 2.4 M IU, IM, weekly, three consecutive weeks |
| 11 | Duncan et al. (2021) | 66, M | Rheumatoid arthritis | Secukinumab (anti-IL17) |  | No | RPR (1/128) | Positive VDRL | C-PenG IV for three weeks (dose not specified) |
|  |  |  |  |  |  |  | FTA-ABS (+) |  |  |
| 12 | Lefeuvre et al. (2021) | 33, M | Multiple sclerosis | Rituximab (anti-CD20) |  | No information | Negative VDRL and TPPA >3 weeks | No information | B-PenG 2.4 M IU, IM, weekly, three consecutive weeks |
|  | Present case | 22, M | Multiple sclerosis | Ocrelizumab (anti-CD20) |  | No new complaints; | VDRL (1/2) | Negative VDRL | B-PenG 2.4 M IU, IM, weekly, three consecutive weeks and Ceftriaxone 2g, IV, daily, 14 days |
|  |  |  |  |  |  | Infection: possible trigger for persistent dysesthesia and reduced right hand muscle strength | CMIA (+) |  |  |

F, Female; M, Male; TNF, Tumor Necrosis Factor; IL, Interleukin; PsO, Psoriasis; PSA, Psoriatic Arthritis; PLEVA, Pityriasis lichenoides et varioliformis acuta; LP, Lymphomatoid papulosis; PCTCL, Primary cutaneous T-cell Lymphoma; HZ, Herpes zoster; VDRL, Venereal Disease Research Laboratory; FTA-ABS, Fluorescent Treponemal Antibody Absorption test; RPR, Rapid Test Reagin; TPHA, Treponema Pallidum Hemagglutination Assay; TPPA, Treponema Pallidum Particle-Agglutination Assay; CMIA, chemiluminescence microparticle immunoassay; C-PenG, Crystalline Penicillin G; B-PenG, Benzathine Penicillin G; M, Millions; IU, International units; IV, Intravenous; IM, Intramuscular:

**References**

1. Bories-Haffner C, Buche S, Paccou J. Secondary syphilis occurring under anti-TNFalpha therapy. Joint Bone Spine. 2010;77:364-5.

2. Asahina A, Ishii N, Tohma S. Secondary syphilis following tumor necrosis factor-α inhibitor treatment for rheumatoid arthritis. J Dermatol. 2012;39:199-201.

3. Bettenworth D, Floer M, Krummenerl T, Wehrmann W, Schärer L, Heidemann J. Advanced-stage syphilis unmasking after immunomodulator therapy in a patient with ulcerative colitis. Am J Gastroenterol. 2012;107:144-5.

4. Assikar S, Doffoel-Hantz V, Sparsa A, Bonnetblanc JM. Early neurosyphilis with etanercept treatment. Eur J Dermatol. 2013;23:901-2.

5. Kase K, Ishii-Osai Y, Sumikawa Y, Yoneta A, Himeno D, Kakutani Y, Yamashita T. Rapidly developed neurosyphilis in a psoriasis patient under treatment with infliximab: A Case Report. Acta Dermato-Venereologica. 2015;95:351-2.

6. Pedrosa AF, Magina S, Azevedo F, Lisboa C. Re-emergence of syphilis in the biological era. Int J Dermatol. 2016;55:e626-e628.

7. Uslu U, Heppt F, Sticherling M. Secondary syphilis infection under treatment with ustekinumab. Clin Exp Dermatol. 2017;42:836-8.

8. Iglesias-Plaza A, Iglesias-Sancho M, Quintana-Codina M, García-Miguel J, Salleras-Redonnet M. Syphilis in the setting of anti-tumor necrosis factor alpha therapy. Reumatol Clin (Engl Ed). 2019;15:e108-e110.

9. Yıldızhan IK, Şanlı HE, Çetinkaya H, Akay BN, Koçyiğit P, Kundakçı N. A rare case of malignant syphilis after adalimumab therapy due to Crohn's disease associated with bariatric surgery. Diagn Microbiol Infect Dis. 2019;95:89-92.

10. Dos Santos CA, Benevides LC, Cardili RN, Pileggi GCS, Ferriani VPL, Roselino AMF. Malignant syphilis in a young patient with juvenile idiopathic arthritis under biological therapy. J Clin Rheumatol. 2021;27:S382-S383.

11. Duncan A, Zingas N, Ahmed A, Shih R. The great imitator: latent neurosyphilis revealed after initiation of the immunosuppressive drug secukinumab. Cureus. 2021;13:e18462.

12. Lefeuvre C, Croué A, Abgueguen P, Letzelter M, Ducancelle A, Grange P, Benhaddou N, Dupin N, Le Guillou-Guillemette H, Le Clec'h C. Serological diagnosis of secondary syphilis in a Rituximab-treated patient: an emerging diagnostic challenge? J Eur Acad Dermatol Venereol. 2021;35:e350-e352
